# Long term efficacy of WT1 peptide vaccine in patients with WT1 overexpressing hematopoeitic and solid malignancies

**DOI:** 10.1186/2051-1426-1-S1-P223

**Published:** 2013-11-07

**Authors:** Anne Letsch, Carmen Scheibenbogen, Antonia Busse, Sebastian Ochsenreither, Igor Wolfgang Blau, Claudia Baldus, Eckhard Thiel, Ulrich Keilholz

**Affiliations:** 1 Hematology, Oncology, and Tumor Immunology, Charité Universitätsmedizin, Berlin, Germany; 2 Institue of Medical Immunology, Charité Universitätsmedizin, Berlin, Germany

## 

We have performed two separate phase II peptide vaccine trials in patients with AML/MDS and in patients with WT1 overexpressing solid tumors with identical vaccine schedule. Here we concentrate on patients remaining on vaccination for at least one year in order to analyze characteristics for long-term vaccination success. Factors considered were disease type, intensity of prior chemotherapy, tumor load and toxicity. Patients with AML and high-risk MDS (n=25) as well as with ovarian (n=7), thyroid (n=2), breast (n=1), gastric (n=1), and larynx cancer (n=1), astrocytoma (n=1) and mesothelioma (n=4) received 0.2mg HLA-A*0201-restricted WT1.126-134 peptide admixed with 1 mg KLH (day 3), and 62.5µg GM-CSF (day 1-4) every 2 weeks x 4, followed by either biweekly or monthly for 10 months and at increased intervals of up to 3-monthly thereafter. Standard criteria were used for response assessment. Patients remained on study in absence of limiting toxicity and disease progression.Toxicity to treatment was mild, and no patient went off protocol for adverse events or consent withdrawal. Eleven patients remained on study for more than 1 year with disease control, including 3/19 patients with active AML, 4/9 patients with AML in CR, 2/7 patients with ovarian cancer and 2/4 patients with mesothelioma. Of these 11 patients 4 remained on study for more than 3 years: A 70 year old man with sAML from MDS, with 5% marrow blasts at baseline and severe anemia after 3 cycles of induction and consolidation chemotherapy, who was 58 months on study. A previously untreated woman with AML with PD and 40% of marrow blasts at baseline, who is 78+ months in SD on study, and a woman with AML in CR after induction and consolidation therapy, who achieved a second CR after initial relapse with 30% marrow blasts during vaccination and 48+ months on study. In addition a man with mesothelioma and PR after initial pleurectomy, who is after 50+ months in SD still being vaccinated. Overall, no correlation between prior treatment and duration on vaccine was apparent, as 2/4 patients in long-term disease control had previously aggressive chemotherapy. Of the solid tumor patients, chemotherapy-pretreated ovarian cancer and mesothelioma appear to be sensitive to WT1 vaccine, although other histologies cannot be excluded due to low patient numbers. These results further underline the potential of WT1 targeting immunotherapies in cancer patients and detailed immunological and molecular analyses might be able to impact on improved vaccine design and to characterize a certain subgroup of patients with specific benefit from such therapies.

